# A Fatal Case of Septic Shock Secondary to *Acinetobacter* Bacteremia Acquired from a Platelet Transfusion

**DOI:** 10.1155/2019/3136493

**Published:** 2019-12-27

**Authors:** C. Nevala-Plagemann, P. Powers, M. Mir-Kasimov, R. Rose

**Affiliations:** ^1^Department of Internal Medicine, University of Utah, Salt Lake City, UT, USA; ^2^Department of Pulmonary Medicine and Critical Care, University of Utah, Salt Lake City, UT, USA; ^3^Department of Medicine, Veterans Affairs Medical Center, Salt Lake City, UT, USA

## Abstract

**Background:**

Transfusion of blood products is a frequent and often necessary lifesaving intervention. While changes to blood bank practices over the past several decades have reduced the infectious complications associated with transfusions, risks still exist. Septic transfusion reactions caused by bacterial contamination of blood products, especially platelets, still occur relatively frequently. Unfortunately, clinical recognition of septic transfusion reactions is difficult due to significant symptom, exam, and laboratory abnormality overlap between different types of transfusion reactions, as well as other conditions. Novel methods have been developed to detect blood product contamination but have yet to be widely implemented in the United States.

**Case Report:**

A 67-year-old male with chronic thrombocytopenia was transfused with platelets prior to a planned procedure. Shortly afterwards, he developed fever and hypotension. He was transferred to the intensive care unit where he was treated with aggressive fluid resuscitation and broad-spectrum antibiotics. The patient went on to develop progressively worsening shock and profound disseminated intravascular coagulation. Blood cultures from the patient and the transfused platelets grew an *Acinetobacter* species. Despite aggressive resuscitative efforts and appropriate antibiotics, the patient died approximately 48 hours following the transfusion reaction.

**Conclusion:**

We report a fatal case of septic shock associated with *Acinetobacter* bacteremia caused by platelet transfusion. Our review of the literature revealed only one other documented platelet transfusion associated fatality caused by *Acinetobacter* species. Novel pathogen reduction and contamination detection methods have been developed but have yet to be widely adopted in the United States.

## 1. Introduction

According to the most recent National Blood Collection and Utilization Survey (NBCUS) and the United States Food and Drug Administration (FDA), there were 11.3 million whole blood and red blood cell transfusions, 2.1 million apheresis platelet transfusions, and 3.6 million plasma transfusions in the United States in 2015 [1]. Given the sheer number of transfusions that occur each year, it is not surprising that despite improvements in blood bank practices over the past few decades, transfusion reactions remain a commonly encountered problem in our medical system.

While many of these transfusion reactions are relatively benign, a recent retrospective analysis of academic centers in the United States found that approximately 1% of all transfusions result in a serious reaction [2]. The most recently published report by the FDA identified 201 deaths that could be attributed to transfusion reactions from 2014 to 2017 [1].

Given the seriousness and frequency of transfusion reactions, prompt and accurate diagnosis is extremely important. This is especially true in cases of septic transfusion reactions, for which prompt initiation of antibiotics is necessary. Unfortunately, the signs, symptoms, and laboratory abnormalities found in septic transfusion reactions share significant overlap with other types of transfusion reactions and other conditions often making diagnosis difficult and delaying appropriate treatment [3, 4].

In this report, we describe a fatal case of septic shock secondary to *Acinetobacter* bacteremia caused by a platelet transfusion. We will discuss the current literature regarding frequency of septic platelet transfusion reactions and will briefly discuss current and future methods being implemented to help prevent these serious reactions from occurring in the future.

## 2. Case Presentation

A 67-year-old male with a history of type II diabetes, chronic obstructive pulmonary disorder, and alcoholic cirrhosis was admitted to our hospital for medical optimization prior to a planned transarterial chemoembolization procedure for recently diagnosed hepatocellular carcinoma. The patient was feeling well at the time of admission with no significant acute complaints. Admission vitals were unremarkable, and his admission labs were notable only for chronic thrombocytopenia with a platelet count of 27,000 per microliter.

In anticipation of his upcoming procedure, two units of apheresis platelets were ordered with a goal of raising his platelet count above 50,000 per microliter to prevent bleeding. Shortly after initiation of the first platelet transfusion, the patient complained of chills and was noted to have a temperature of 100.8 F. The transfusion was stopped, and the patient was administered acetaminophen and diphenhydramine. Approximately 1 hour later, the patient developed tachycardia, tachypnea, and hypotension and was found to have an increased temperature of 104.9 F.

The patient was transferred to the intensive care unit of our facility where he was aggressively fluid resuscitated and started on vasopressors due to persistent hypotension. Broad-spectrum antibiotics were initiated with piperacillin/tazobactam and vancomycin. Within a few hours of admission to the ICU the patient developed severe DIC requiring aggressive blood product replacement and intubation for airway protection due to development of a large hematoma at the site of his right internal jugular central line.

Blood culture results were available approximately 18 hours after the transfusion reaction and revealed bacteremia with a Gram-variable organism. Cultures were grown on MacConkey agar and had variable lactose fermentation. Given morphology and lactose fermentation results, the specimens were sent for immediate matrix-assisted laser desorption/ionization—time of flight (MALDI-TOF), which positively identified them as genus *Acinetobacter*. Cultures were obtained from the unit of platelets, and the patient had been transfused with grew *Acinetobacter* as well, identified via the same methodology. Antibiotic coverage was changed to tobramycin and meropenem to cover possible resistant strains of *Acinetobacter*. Unfortunately, over the subsequent 24 hours, the patient's vasopressor requirements continued to rise. Given his ongoing clinical deterioration, further aggressive treatment was felt to be unlikely to improve his condition. A family meeting was held, care was withdrawn, and the patient expired shortly after. Autopsy revealed the likely cause of death to be septic shock with multiorgan failure. Bacterial susceptibility results available following the patient's death revealed full susceptibility to meropenem but only intermediate susceptibility to piperacillin-tazobactam.

The case was reported to the FDA, and samples were sent for genomic sequencing. Per the FDA, from May 2018 to October 2018, there were four patients from three states who experienced sepsis after platelet transfusions contaminated with *Acinetobacter*. Sequencing demonstrated that both the patient and platelet bag *Acinetobacter* isolates were molecularly related. The platelet donor was investigated, and no *Acinetobacter* isolates were identified in the patient's urine, perianal area, or multiple skin sites. During their investigation, swabs from the platelet agitators at the platelet manufacturing facility, as well as the hospital platelet agitator, identified *Acinetobacter* [5].

## 3. Discussion

Around two million platelet transfusions are performed each year in the United States [6]. While platelet transfusions are often necessary to prevent life-threatening bleeding in thrombocytopenic patients, they do come with a risk. Septic transfusion reactions caused by bacterial contamination of blood products have long been known to be a problem, especially with transfusion of platelets due to their storage at room temperature. In fact, the bacterial contamination rate of apheresis platelet has been shown to be around 1 in 5000 transfusions with a risk of transfusion-associated sepsis around 1 in 100,000 transfusions [6].

Death related to transfusion of contaminated platelets, however, remains rare. From 2012 to 2015, there were only 10 reported fatalities related to bacterial contamination of platelets in the United States. Furthermore, infection and death related to *Acinetobacter* species, as was the case in this patient, is exceedingly rare. Prior to the four cases in 2018, only one other case had been reported by the FDA in 2013 [1]. *Acinetobacter* species are Gram-negative bacteria that possess inherent resistance to desiccation, allowing them to persist on environmental surfaces [7]. This fact, coupled with a natural predilection to virulence factors that allow immune evasion and high frequency of extreme drug resistance, makes *Acinetobacter* a formidable organism.

The finding of a potentially multidrug-resistant pathogen as the causative organism in this case and the fact that this patient died despite relatively prompt diagnosis and appropriate antibiotic treatment reveals the need for further implementation of transfusion-associated infection prevention methods. In 2004, the AABB introduced standard 5.1.5.1, requiring the blood collection industry to implement measures to detect and limit bacteria in platelet components [8]. This includes screening of the donor for symptoms that may indicate septicemia, proper skin disinfection prior to venipuncture, diversion of the first aliquot of collected blood, and cultures at time of apheresis. This resulted in a 50–75% reduction in septic transfusion reactions [9]. At our facility, once received, the platelets undergo a visual inspection, and those with evidence of swirling or visual contamination are discarded. In this case, the contaminated platelet concentrate (PC) had documented negative cultures at 24 hours and a negative visual inspection prior to release to the patient. Other methods such as pH, glucose levels, and Gram-stain coloration have been shown to be of low sensitivity [10].

Several methods are currently being developed and utilized in facilities to reduce the number of transfusion-transmitted bacterial infections (TTBIs) and can broadly be split into secondary detection and pathogen reduction. Of the secondary detection methods, there are currently two antigen-based tests approved by the FDA. The first, the Platelet Pan Genera Detection (PGD) test, developed by Verax Biomedical, detects either lipoteichoic acid in Gram-positive bacteria, or lipopolysaccharide in Gram-negative bacteria [11]. The second, the BacTx assay, developed by Immunetics, Inc., detects bacterial peptidoglycan [12]. These tests have been demonstrated to both increase bacterial detection, and in so doing potentially increase platelet shelf life to 7 days [13]. Other groups have examined the utility of secondary bacterial cultures. Bloch et al. implemented secondary bacterial cultures in their standard testing for PCs and were able to detect 8 contaminated platelets that would have otherwise been transfused out of a total of 23,044 PCs [14].

The competing strategy to address contaminated platelets is pathogen reduction. Currently, there are three main technologies, which use a photochemical approach for the reduction of potential pathogens [15]. The THERAFLEX system developed by Macopharma and the German Red Cross Blood Service uses UVC light to form pyrimidine dimers that block the elongation of nucleic acid transcripts. The INTERCEPT (Cerus Corporation) and MIRASOL (TerumboBCT) systems utilize UVA/B light in concert with either psoralen or riboflavin, respectively, to crosslink DNA and reduce bacterial, viral, and parasitic load in treated products. The INTERCEPT method is presently the only method that is approved by FDA for pathogen reduction of platelets in the United States.

While the use of secondary detection or pathogen reduction methods has been demonstrated to be effective in reducing TTBIs, they can be costly and work-intensive. Prior to the implementation of these methods, a cost analysis is a prudent exercise, especially in large transfusion centers. In one example, Li et al. compared platelets using PRT (INTERCEPT) and those using secondary testing (PGD) and found that PRT was significantly more costly and did not reliably extend shelf life to 7 days [16]. An exhaustive comparison between methodologies is beyond the scope of this article, but these and other studies highlight the importance of a critical evaluation of institutional platelet transfusion practices. In September 2019, the FDA released an updated bacterial risk control strategy to continue reducing the number of TTBIs [17]. They divided PCs into two main classes: (1) apheresis platelets and/or prestorage pools of WBD platelets and (2) single units and poststorage pools of WBD platelets. Specific recommendations were given for each group and warrant detailed review by individual institutions. Broadly, the FDA has begun to recommend secondary detection including rapid testing and secondary culture, as well as pathogen reduction.

## 4. Conclusion

While transfusion reaction due to bacterial contamination is rare, it is an important complication to consider when a reaction occurs. Newer methods of detection of bacterial contamination may help reduce transfusion complications.
